# Supplementary material for: Menopausal Status Contributes to Overall Survival in Endometrial Cancer Patients
Source: Cancers (Basel). 2023 Jan 10;15(2):451. doi: 10.3390/cancers15020451 (PMC9856958; doi:10.3390/cancers15020451)
Supplement: Supplementary file 1 [file cancers-15-00451-s001.zip › cancers-1989741-supplementary.pdf]

Supplementary materials

Menopausal status contributes to overall survival in endometrial cancer patients

Bartłomiej Barczyński[1,*], Karolina Frąszczak[1], Artur Wnorowski[2], Jan Kotarski[3]

[1] 1st Chair and Department of Oncological Gynaecology and Gynaecology, Medical University in Lublin, Poland

[2] Department of Biopharmacy, Medical University in Lublin, Poland

[3] Independent Laboratory of Cancer Diagnostics and Immunology, Medical University in Lublin, Poland

* Correspondence: bbarczynski@poczta.onet.pl; Tel.: +48504108558

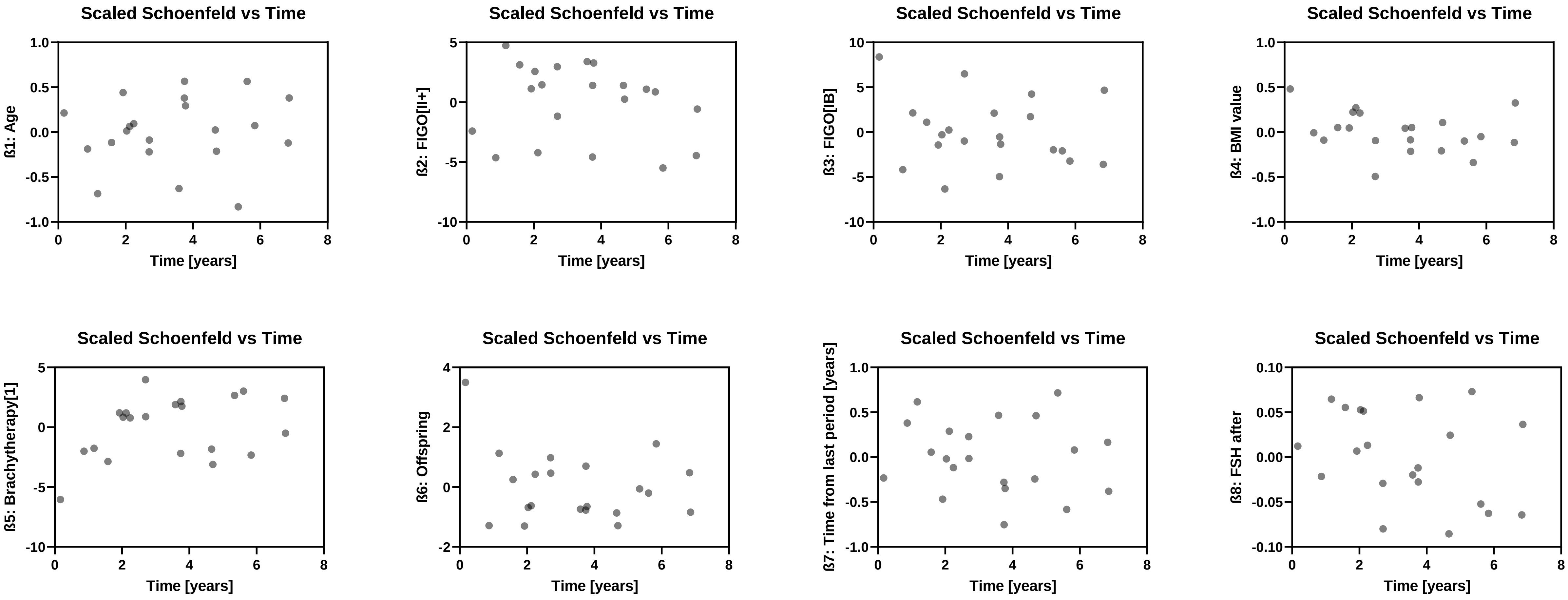

Figure S1. Scaled Schoenfeld residuals plotted against time.

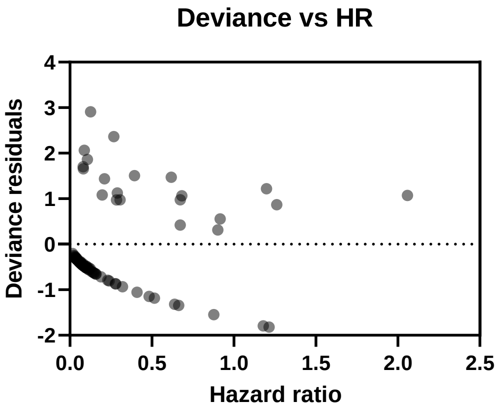

Figure S2. Deviance residuals plotted against hazard ratio. The large dense cluster of points in the lower left region of the graph is due to a high proportion of censored observations in the data set. By definition, censored observations must have negative deviance residuals.

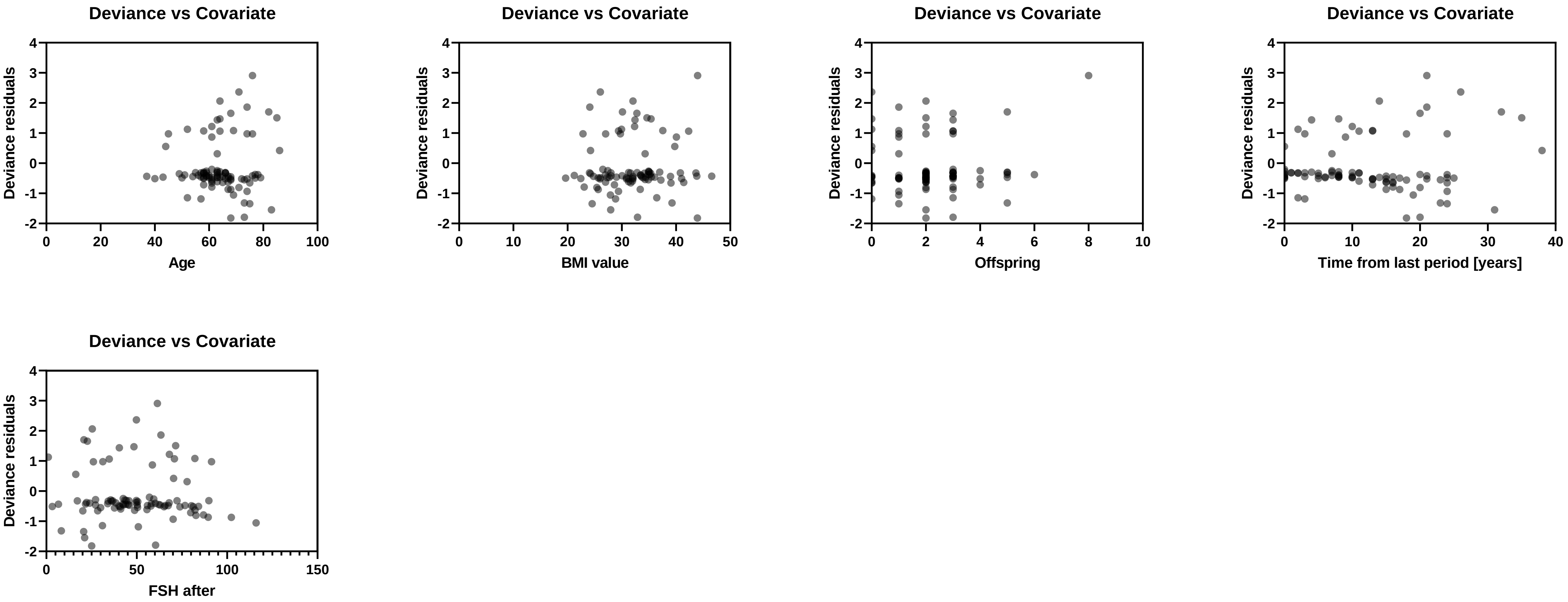

Figure S3. Deviance residuals plotted against hazard ratio. The large dense cluster of points in the lower left region of the graph is due to a high proportion of censored observations in the data set. By definition, censored observations must have negative deviance residuals.
